# Surgical strategies and long-term survival for third ventricle chordoid gliomas: a systematic review and clinical algorithm

**DOI:** 10.1007/s10143-026-04375-x

**Published:** 2026-06-25

**Authors:** Omar Alomari, Beyzanur Güney, Irem Uslu, Umid Sulaimanov, Yerkebulan Serikkanov, Nafiye Sanlier, Oyku Ozturk, Abdullah Keles, Ufuk Erginoglu, Brian Snyder, Ankush Bhatia, Mustafa K. Baskaya

**Affiliations:** 1 Hamidiye International School of Medicine, University of Health Sciences, Istanbul, 3400 Türkiye; 2 Faculty of Medicine, Pamukkale University, Denizli, Turkey; 3 Department of Neurosurgery, University of Wisconsin School of Medicine, Madison, WI USA; 4 Department of Neurology, University of Wisconsin School of Medicine, Madison, WI USA; 5 Department of Neurological Surgery, School of Medicine and Public Health, University of Wisconsin, Madison CSC K4/822, 600 Highland Avenue, Madison, WI 53792 USA

**Keywords:** Chordoid glioma, Hypothalamic morbidity, Neurosurgical oncology, PRKCA mutation, Radiosurgery, Third ventricle

## Abstract

Chordoid gliomas are rare World Health Organization Grade II neoplasms of the third ventricle. While Gross Total Resection (GTR) has traditionally been the primary surgical objective, the intimate adherence to the hypothalamus and optic apparatus of these tumors creates a therapeutic dilemma for balancing oncological control against the risk of severe neurological and endocrine morbidity. This study aims to guide optimal management by bridging the evidence gap with the largest systematic review to date, analyzing clinical characteristics, surgical outcomes, and survival data. A systematic review was conducted according to PRISMA 2020 guidelines, searching Web of Science, PubMed, Scopus, and Embase for studies from database inception to November 2025. Data included patient demographics, clinical presentation, radiological phenotypes, surgical techniques, molecular profiles, and follow-up outcomes. Kaplan-Meier survival estimates and log-rank tests were used to assess survival outcomes by extent of resection. All analyses were performed using R-software (version 4.3.1). The cohort (N = 198; mean age 41.8 years; female-to-male ratio 2:1) predominantly presented with headache (51.3%), visual disturbances (37.5%), and cognitive deficits (24.4%). GTR was achieved in 56% of patients, while 32% underwent Subtotal Resection (STR), and 10% biopsy only. Kaplan-Meier analysis revealed a significant survival advantage for GTR, with a stable 5-year survival rate of 91.9% compared to 54.7% for STR (p = 0.0089). Molecular profiling identified PRKCA D463H as the predominant driver mutation, with BRAF V600E observed in a minority of cases. GTR is associated with superior long-term survival in the literature and may be considered when anatomically feasible. However, because this association may be confounded by tumor adherence and surgical selection, resection strategies must be strictly individualized to balance tumor control against hypothalamic morbidity.

**Supplementary Information:**

The online version contains supplementary material available at 10.1007/s10143-026-04375-x.

## Introduction

Chordoid gliomas of the third ventricle are rare, low-grade neuroepithelial tumors of the central nervous system. These were first defined as a distinct clinicopathological entity by Brat et al. in 1998 and currently classified as World Health Organization (WHO) Grade II neoplasms [1, 2]. These tumors typically arise from the anterior wall or roof of the third ventricle, typically near the lamina terminalis and hypothalamus [3].

Epidemiologically, chordoid gliomas occur predominantly in adults, peaking in the fourth and fifth decades of life, with a female-to-male ratio of approximately 2:1 [4]. Clinically, due to their central diencephalic location, these often present with symptoms related to mass effect, with common presentations including obstructive hydrocephalus (headache, nausea, papilledema), followed by visual disturbances due to optic chiasm compression and endocrine dysfunction resulting from hypothalamic-pituitary axis involvement [5]. Pathologically, these are epithelioid cell clusters within a mucinous stroma, with a unique immunohistochemical profile consisting of a diffuse expression of thyroid transcription factor-1 (TTF-1) and glial fibrillary acidic protein (GFAP), which distinguishes them from other third ventricular masses, (e.g. craniopharyngiomas or meningiomas) [6, 7]. Bielle et al. proposed that chordoid gliomas share immunophenotypic features with the organum vasculosum of the lamina terminalis (OVLT), supporting a distinct embryological origin [6].

Surgical resection remains the primary treatment, aiming to relieve hydrocephalus and achieve oncological control [8]. Radiological diagnosis is often suggested by a solid, well-circumscribed, round-to-ovoid mass that is isointense on T1-weighted MRI and vividly enhancing after contrast administration [9].

Gross total resection (GTR) provides the highest probability of progression-free survival, with very low recurrence rates reported for GTR cases [10]. Various surgical corridors have been utilized, including transcallosal, transcortical, and trans-lamina terminalis, chosen based on the tumor’s vertical extension and relationship to the Foramen of Monro [11].

Despite defined histopathology, optimal management strategy remains controversial due to the tumor’s rarity and high risks associated with its location. GTR is frequently complicated by tight adherence to vital neurovascular structures, particularly the hypothalamus and optic apparatus. Consequently, aggressive GTR attempts may result in severe postoperative morbidity, including permanent diabetes insipidus, hypothalamic obesity, and severe short-term memory deficits [12, 13].

There is currently no consensus on whether subtotal resection (STR) followed by adjuvant therapy is superior to aggressive GTR for preserving quality of life (QoL). Furthermore, the role of adjuvant therapies such as Gamma Knife Radiosurgery (GKRS) or fractionated stereotactic radiotherapy remains ill-defined. Most available data derive from case reports and small series, limiting predictive modeling and standardized follow-ups [14].

Our study aims to address this gap by providing a comprehensive systematic review of third ventricle chordoid gliomas. By pooling all reported cases in the literature, we assembled the largest dataset to date. Our objective is to statistically analyze pooled data to evaluate the safety and efficacy of different surgical strategies and adjuvant therapies, ultimately providing evidence-based guidance to optimize the balance between oncological control and functional preservation.

## Materials & Methods

We conducted a systematic literature review to evaluate the clinical characteristics, radiological features, surgical management, and survival outcomes for third ventricle chordoid gliomas, adhering to the Preferred Reporting Items for Systematic Reviews and Meta-Analysis (PRISMA 2020) guidelines to ensure methodological rigor and consistency [15]. This study was registered in the International Prospective Registry of Systematic Reviews database prior to publication selection and data extraction (www.crd.york.ac.uk/Prospero/, PROSPERO ID: CRD420251251487. Human ethics and consent to participate declarations, and clinical trial numbers, are not applicable for this literature review.

### Search strategy

A comprehensive search was independently performed by two reviewers using Web of Science, Medline (via PubMed), Scopus, and Embase. All eligible studies published from database inception to November 2025 were included. The search strategy incorporated relevant keywords and Medical Subject Headings (MeSH), including “chordoid glioma”, “third ventricle tumors”, “hypothalamic glioma”, and “chordoid neoplasm”. Terms were combined using Boolean operators “OR” and “AND”, following the Cochrane Handbook for Systematic Reviews [16]. Manual searches of reference lists were also conducted to identify additional studies. Supplementary Table 1 provides detailed search terms and results for each database.

## Eligibility criteria

This review focused on studies involving patients with pathologically-confirmed chordoid glioma diagnoses. Inclusion criteria encompassed randomized controlled trials (RCTs), prospective and retrospective cohort studies, case series, and case reports. The target population consisted of human subjects of any age or sex diagnosed with WHO Grade II chordoid glioma. Eligible studies had to report specifics of clinical presentations, radiological findings, surgical approaches, extent of resection, and/or survival outcomes. Studies were excluded if they were reviews, editorials, or meeting abstracts that lacked original patient data. Also excluded were animal or in vitro experimental models studies, and articles published in languages other than English, unless complete translations were available.

## Study selection and data extraction

After duplicate reports were removed, two independent reviewers (B.G., I.U.) screened the titles and abstracts. Subsequently, two additional reviewers (O.A., U.S.) assessed the full-text articles for eligibility using the predefined inclusion/exclusion criteria. Any disagreements at the abstract screening stage were resolved by discussion or by a third reviewer (O.A.).

Primary data extraction was conducted independently by two reviewers (O.A., B.G.) using a pre-piloted Excel spreadsheet developed explicitly for this review. An additional reviewer (I.U.) subsequently verified extracted data for accuracy and consistency. Data was extracted as discrete events and where applicable, continuous variables were reported using means and standard deviations.

## Risk of bias assessment

Two independent reviewers (I.U., B.G.) assessed bias risk for in all included studies. Case reports and case series were evaluated with the Joanna Briggs Institute (JBI) critical appraisal tool, comprising eight questions assessing study design, execution, and reporting, and ability to minimize bias [17]. For cohort and cross-sectional studies, The National Institutes of Health (NIH) quality assessment tool was applied, consisting of 14 questions related to research methodology, sample representativeness, and confounding control [18]. Each question was answered with yes, no, or unclear, with any discrepancies between reviewers resolved through discussion.

## Grading of evidence and levels of recommendations

Included studies were assessed according to the American Association of Neurological Surgeons and the Congress of Neurological Surgeons (AANS/CNS) grading criteria for medical evidence quality and strength of clinical recommendations [19]. Two independent reviewers (O.A., B.G.) evaluated each study, with any disagreements resolved via discussion. The class of evidence (Class I, II, III) was determined based on cohort size, outcome measures, and follow-up duration. The corresponding strength of recommendations (Levels I-III) was then assigned based on the highest level of supporting evidence.

### Statistical analysis

Descriptive statistics were used to summarize pooled data; continuous variables (e.g., age) were reported as means with ranges, while categorical variables (e.g., symptoms, surgical approaches) were reported as frequencies and percentages. Survival analysis was performed using the Kaplan-Meier method to estimate overall survival (OS) at 1, 3, and 5 years. The Log-Rank test compared survival outcomes between subgroups, specifically between those undergoing GTR and STR, with *p* < 0.05 considered statistically significant. All statistical analyses and figure generation were conducted using R software (*Version 4.3.1*) using the “ggplot2” and “survival” packages.

## Results

### Study characteristics & patient demographics

**Fig. 1:**
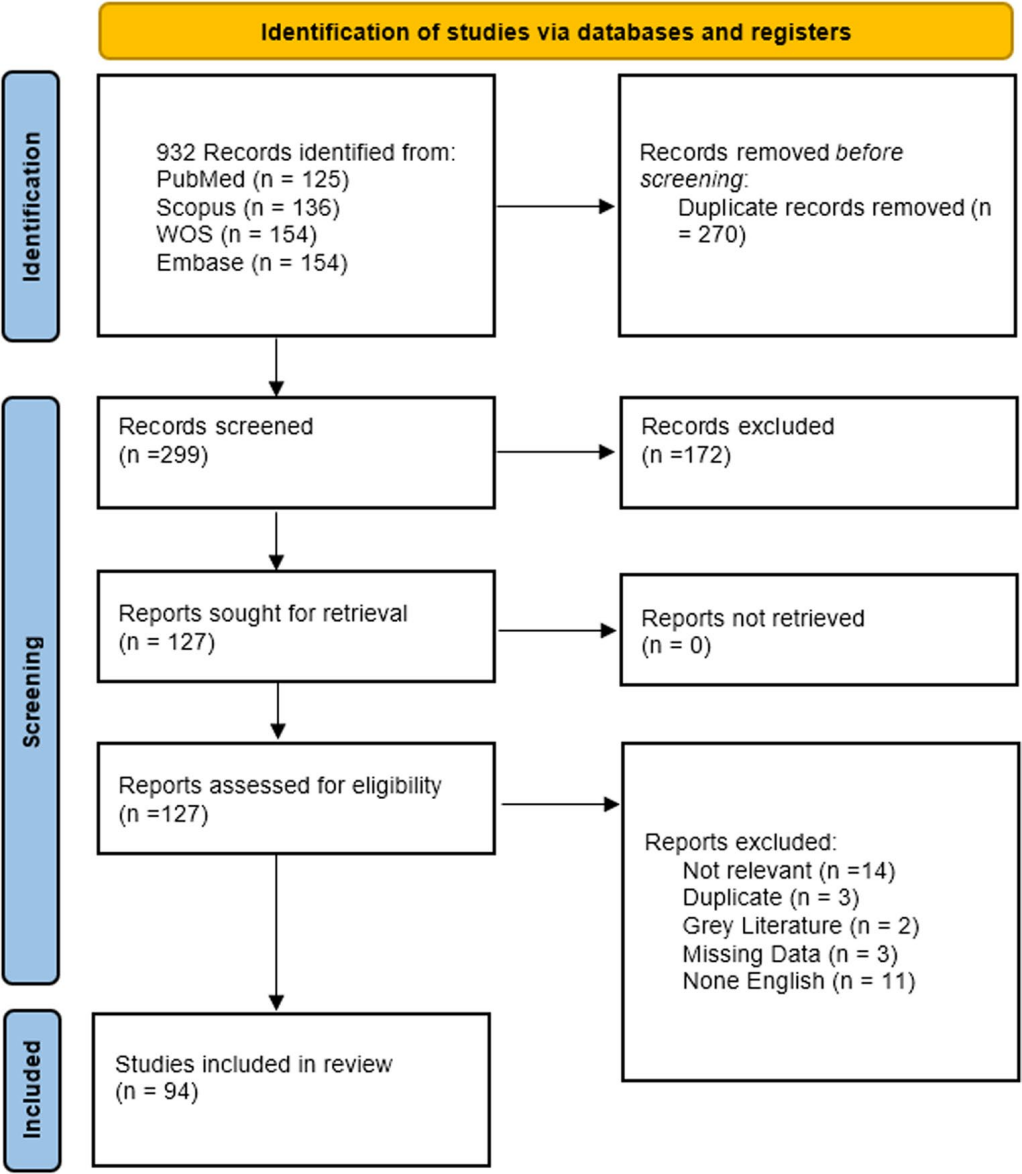
PRISMA flow diagram of the search and selection process for included articles. Flowchart details the systematic search strategy and selection phases from identification, through screening to the final studies included in the review

This systematic review included 94 studies comprising 198 patients diagnosed with chordoid glioma of the third ventricle screened from 299 publications (Fig. 1) [1, 3, 4, 6–8, 10, 12, 20–94]. These studies were predominantly retrospective, primarily comprising single case and small series reports, with few retrospective cohort studies (Table 1). These third ventricle tumors often involved suprasellar and hypothalamic regions. Cases were distributed worldwide, with significant USA, China, Japan, South Korea, and European contributions. Mean patient age was 41.83 years (range 7–72 years). Females comprised 58% of the 192 patients with available gender data.

**Table 1:** Baseline Characteristics of the Included Studies

Study ID	Year	Country	Study Design	Sample size	Age	Sex	Location	Presenting Symptoms	CNS Grading	Quality Assessment
Brat et al.	1998	USA	Case Series	8	50, 70, 59, 47, 31, 56, 31, 35	7 F, 1 M	Third Ventricle (x2) Suprasellar & Third Ventricle (x5) Hypothalamic Region & Third Ventricle (x1)	Lethargy, urinary incontinence, ataxia; Progressive ataxia; Obstructive hydrocephalus; Headache, nausea, vomiting; Hypothyroidism, diabetes insipidus; GI symptoms, weight loss; Amenorrhea, psychotic disorder	Level IV	High Quality
Cenacchi et al.	1999	Italy	Case Series	3	34, 40, 43	2 M, 1 F	Hypothalamic & Third Ventricle	Not specified in provided text	Level IV	Fair Quality
Reifenberger et al.	1999	Germany	Case Series	5	56, 31, 53, 65, 35	3 F, 2 M	Suprasellar & Third Ventricle (x3)Hypothalamic Region (x1)Third Ventricle (x1)	Headaches, fatigue, flimmer scotoma; Visual disturbances, weight gain, bitemporal hemianopia; Speech problems, facial weakness; Organic psychosyndrome	Level IV	High Quality
Vajtai et al.	1999	Switzerland	Case Report	1	60	F	Third Ventricle	Headache, memory impairment, somnolence	Level V	High Quality
Tonami et al.	2000	Japan	Case Report	1	42	F	Third Ventricle	1-year history of amenorrhea, memory impairment, short periods of unconsciousness	Level V	High Quality
Sanchez et al.	2000	USA	Case Report	1	36	M	Suprasellar & Third Ventricle	Diminished visual acuity (left eye), left supero-temporal visual field defect	Level V	High Quality
Cenacchi et al.	2001	Italy	Case Series	3	34, 40, 43	2 M, 1 F	Third Ventricle	Not specified in provided text	Level IV	Fair Quality
Galloway et al.	2001	UK	Case Report	1	54	M	Hypothalamic & Suprasellar	Sudden onset of left-sided weakness, ataxia, deteriorating visual acuity	Level V	High Quality
Hanbali et al.	2001	USA	Case Report	1	57	M	Lamina Terminalis	Worsening headache, generalized malaise, anorexia, loss of olfactory sensation	Level V	High Quality
Grand et al.	2002	France	Case Report	1	41	F	Lamina Terminalis	Visual disturbance, headache	Level V	High Quality
Lee et al.	2002	Korea	Case Report	1	48	F	Sellar & Suprasellar	1-year history of headache and dizziness	Level V	High Quality
Pasquier et al.	2002	France	Case Report	2	35, 39	1 M, 1 F	Third Ventricle (Anterior)	18-month history of headaches, nausea, insomnia, visual disturbances; Headaches, diplopia, bitemporal hemianopsia	Level V	High Quality
Nakajima et al.	2003	Japan	Case Report	1	49	F	Suprasellar & Third Ventricle	1-year progressive headache, 2-month memory impairment, urinary incontinence	Level V	High Quality
Raizer et al.	2003	USA	Case Report	1	57	F	Third Ventricle (Anterior)	Driving difficulties, episodes of syncope (“drop attacks”)	Level V	High Quality
Sanchez et al.	2003	USA	Case Report	1	12	M	Hypothalamic Region	Bilateral blurred vision, left optic neuropathy	Level V	High Quality
Sato et al.	2003	Japan	Case Report	1	65	F	Third Ventricle (Anterior)	2-month history of headache	Level V	High Quality
Suh et al.	2003	Korea	Case Report	1	48	F	Sellar & Suprasellar	1-year history of headache and dizziness, bitemporal hemianopsia	Level V	High Quality
Taraszewski et al.	2003	Korea	Case Report	2	62, 51	1 M, 1 F	Lamina TerminalisThird Ventricle	Headache, polydipsia, polyuria; Hypersomnia, visual deterioration	Level V	High Quality
Buccoliero et al.	2004	Italy	Case Report	1	56	F	Lamina Terminalis	Incidental finding after fall (cranial trauma)	Level V	High Quality
Kurian et al.	2005	The UK	Case Report	2	32, 37	2 F	Suprasellar & Third VentricleSuprasellar	Visual symptoms (progressive deterioration left eye), headache, amenorrhoea, weight gain; Acute hydrocephalus, memory disturbance, confusion, visual field defect	Level V	High Quality
Baehring et al.	2006	USA	Case Report	1	71	F	Suprasellar	Difficulty reading (visual deterioration over years), heteronymous inferior quadrantanopsia	Level V	Fair Quality
Jung et al.	2006	Korea	Case Report	1	50	F	Suprasellar & Third Ventricle	Cognitive dysfunction for 4 years, hyperphagia, memory problems, weight gain	Level V	High Quality
Leeds et al.	2006	USA	Case Report	1	57	M	Suprasellar	General fatigue, severe headaches, malaise, anorexia, loss of smell, increased sweating	Level V	High Quality
Nga et al.	2006	Singapore	Case Report	1	49	F	Third Ventricle (Anterior)	Drowsiness, slowness of movement, inability to ambulate, blurred vision	Level V	High Quality
Takei et al.	2006	USA	Case Report	1	42	F	Third Ventricle (Diencephalon)	Change in ability to function for 6 months, amnestic, confused	Level V	High Quality
Chung et al.	2007	Korea	Case Report	1	48	M	Midbrain & Third Ventricle	Progressive headache, memory impairment, gait disturbance, diplopia	Level V	High Quality
Carrasco et al.	2008	Spain	Case Report	1	53	F	Third Ventricle	Sudden visual disturbances, confusion, dizziness, superior bitemporal quadrantanopsia	Level V	High Quality
Jain et al.	2008	India	Case Report	2	7, 55	1 M, 1 F	Thalamic RegionThird Ventricle (Anterior)	Fever, headache, vomiting, right facial paresis, limb weakness; Memory impairment, urinary incontinence, seizures	Level V	Fair Quality
Vanhauwaert et al.	2008	Belgium	Case Report	1	58	F	Third Ventricle	Progressive memory disturbances, inattention, hypersomnia	Level V	High Quality
Dziurzynski et al.	2009	USA	Case Report	1	41	F	Suprasellar & Third Ventricle	Progressive mental status changes, personality changes, weight gain, temperature dysregulation, polyuria/polydipsia	Level V	High Quality
Horbinski et al.	2009	USA	Case Series	5	41, 36, 36, 50, 72	3 M, 2 F	Third Ventricle (x3)Suprasellar & Third Ventricle (x2)	Cognitive deterioration, stupor; Headaches, nausea, blurred vision; Progressive visual symptoms; Lethargy, urinary incontinence, ataxia; Syncopal episodes, cognitive difficulties	Level IV	High Quality
Iwami et al.	2009	Japan	Case Report	1	61	F	Third Ventricle (Anterior)	History of syncope	Level V	High Quality
Kawasaki et al.	2009	Japan	Case Report	2	42, 51	1 M, 1 F	Third Ventricle (Anterior)	Severe headache, deterioration of consciousness, decline of visual acuity; Visual disturbance	Level V	High Quality
DeSouza et al.	2010	The UK	Case Report	1	48	F	Third Ventricle (Floor)	5 months of fluctuating visual failure, progressive headaches	Level V	High Quality
Kim et al.	2010	Korea	Case Report	1	27	F	Thalamus	Worsening headache, visual disturbance, left temporal visual field defect	Level V	High Quality
Hinai et al.	2011	Canada	Case Report	1	50	F	Third Ventricle (Anterior)	Short-term memory loss, occasional headaches	Level V	High Quality
Liu et al.	2011	China	Case Report	1	45	F	Suprasellar & Third Ventricle	Intermittent headache (10yrs), memory impairment, lethargy (1 year)	Level V	High Quality
Vij et al.	2011	India	Case Report	1	48	M	Third Ventricle	Gradual vision loss, focal seizures, incontinence, somnolence	Level V	High Quality
Bastin et al.	2012	Belgium	Case Report	1	31	M	Third Ventricle (Posterior)	Severe headaches, nausea, right arm paresthesia	Level V	High Quality
Can et al.	2012	Turkey	Case Report	1	37	M	Third Ventricle (Anterior)	10-day left-side weakness, temporary headaches, memory impairment	Level V	High Quality
Ghosal et al.	2012	India	Case Report	1	48	M	Suprasellar & Third Ventricle	Decreased speech output, inability to stand/walk, urinary incontinence	Level V	High Quality
Romero-Rojas et al.	2012	Colombia	Case Report	1	39	M	Third Ventricle (Anterior)	Headache, nausea, progressive ataxia, hydrocephalus	Level V	High Quality
Sanches et al.	2012	Brazil	Case Report	1	59	F	Third Ventricle (Septum Pellucidum)	Moderate holocranial headache	Level V	High Quality
Scheurkogel et al.	2012	The Netherlands	Case Report	1	30	M	Suprasellar	Longstanding visual disturbances, worsened over last month	Level V	High Quality
Xian et al.	2012	Turkey	Case Report	1	53	F	Suprasellar & Third Ventricle	Decreased vision, nasal visual field deficit, headache	Level V	High Quality
Kobayashi et al.	2013	Japan	Case Series	3	55, 31, 61	3 F	Third VentricleSuprasellar (x2)	Nausea, hypertension; Deterioration of myopia; Loss of consciousness, amblyopia	Level IV	High Quality
Ni et al.	2013	China	Case Series	4	41, 25, 35, 57	4 F	Suprasellar & Third VentricleThird Ventricle (Anterior) (x3)	Intermittent headache, dizziness; Nausea/vomiting, decline in visual acuity; Amenorrhea, progressive visual deterioration; Drowsiness, near amnesia	Level IV	Fair Quality
Al-Zubidi et al.	2014	USA	Photo Essay	1	37	M	Third Ventricle (Anterior-Inferior)	Progressive “hazy vision”, bitemporal hemianopia	Level V	High Quality
Khedaoui et al.	2014	Spain	Case Report	1	54	F	Third Ventricle	Headaches and facial paresthesia	Level V	Fair Quality
Michotte et al.	2014	Belgium	Case Report	1	48	F	Suprasellar	Progressively worsening memory problems, depression, urinary incontinence	Level V	High Quality
Tanboon et al.	2014	Thailand	Case Report	1	29	M	Suprasellar & Third Ventricle	Memory impairment, somnolence, lethargy	Level V	High Quality
Bielle et al.	2015	France	Retrospective Cohort	13	42, 45, 27, 56, 39, 46, 71, 65, 34, 70, 60, 68, 33	6 M, 7 F	Third Ventricle (Floor/Anterior/General)	Symptoms not specified in source	Level III	High Quality
Bongetta et al.	2015	Italy	Case Report	1	43	F	Midbrain/Third Ventricle	Headache, asthenia, mood depression, papilledema	Level V	High Quality
Bora et al.	2015	India	Case Report	1	18	M	Suprasellar & Third Ventricle	Severe bifrontal headache with vomiting, transient loss of consciousness	Level V	High Quality
Destefani et al.	2015	Brazil	Case Report	1	27	M	Third Ventricle	Headaches, memory loss, weight gain, hyperphagia, behavior changes	Level V	High Quality
Hewer et al.	2015	Switzerland	Case Report	1	52	M	Suprasellar	Visual disturbances	Level V	Fair Quality
Morais et al.	2015	Brazil	Case Report	1	13	F	Third Ventricle (Floor)	Progressive intermittent holocranial headaches	Level V	High Quality
Qixing et al.	2015	China	Case Report	2	48, 27	1 M, 1 F	Suprasellar & Third Ventricle	Progressive visual failure, decreased binocular vision; Progressive headache and vision loss	Level V	High Quality
Thavaratnam et al.	2015	Singapore	Case Report	1	30	F	Suprasellar & Third Ventricle	Problems with peripheral vision, bitemporal hemianopia	Level V	High Quality
Calanchini et al.	2016	The UK	Case Report	1	48	F	Third Ventricle (Floor)	Mild fatigue, thirst, polydipsia, polyuria, nocturia, intermittent morning headache, SIADH	Level V	High Quality
Carretero et al.	2016	Spain	Case Report	1	30	M	Suprasellar & Third Ventricle	Hypaesthesia/paresthesia right face, mild dysarthria, mild headache, homonymous inferior quadrantanopia	Level V	High Quality
Ki et al.	2016	Korea	Case Report	1	34	M	Suprasellar & Third Ventricle	10-day headache	Level V	High Quality
Oh et al.	2016	Korea	Case Series	6	Not specified	Not specified	Not specified	Not specified (Abstract of 6 cases)	Level IV	Low Quality
Poyuran et al.	2016	India	Case Report	1	45	M	Suprasellar & Third Ventricle	Multiple generalized tonic-clonic seizures, increasing memory loss	Level V	High Quality
Zeinalizadeh et al.	2016	Iran	Case Report	1	43	F	Suprasellar & Third Ventricle	Headache, somnolence, decreased vision	Level V	High Quality
Cunha et al.	2017	Portugal	Case Report	1	71	M	Suprasellar	Progressive visual loss (right eye), right temporal hemianopia	Level V	High Quality
Erwood et al.	2017	USA	Case Report	1	46	F	Third Ventricle (Floor)	Progressive headache, neurocognitive changes, nausea/vomiting, fatigue, weight loss, short-term memory loss	Level V	High Quality
García-García et al.	2017	Spain	Case Report	1	46	F	Suprasellar	Amenorrhea, progressive decrease in visual acuity & libido, inferior bitemporal quadrantanopsia	Level V	High Quality
Yao et al.	2017	China	Case Report	2	45, 38	1 M, 1 F	Suprasellar & Third Ventricle	Memory decline, sleepiness, dizziness, headache, visual blurring; 10-day history of headache	Level V	High Quality
Danilowicz et al.	2018	Argentina	Case Series	2	18, 46	2 F	Sellar & Suprasellar (ext. to V3)	Intense headaches, nausea, vomiting, visual abnormalities, sleepiness, amenorrhea; Amenorrhea & galactorrhea, worsening headache	Level IV	High Quality
Dogan et al.	2018	USA	Case Report	1	42	F	Suprasellar & Third Ventricle	Sudden severe headache, depressed consciousness; History of excessive urination, memory problems	Level V	High Quality
Estronza et al.	2018	Puerto Rico	Case Report	1	42	M	Suprasellar & Third Ventricle	Acute-onset major depressive disorder with psychosis, confusion, agitation, hyponatremia, seizure	Level V	High Quality
Huo et al.	2018	Australia	Case Report	1	37	F	Third Ventricle	Acute severe generalized headache, nocturia	Level V	High Quality
Lisievici et al.	2018	Romania	Case Report	1	57	M	Suprasellar	Signs of elevated intracranial pressure	Level V	Fair Quality
Shinohara et al.	2018	Japan	Case Report	1	46	F	Lamina Terminalis	Mild headache (6mo)	Level V	High Quality
Johannes et al.	2019	Germany	Retrospective Cohort	3	35, 67, 44	1 M, 2 F	Third Ventricle	Chronic headache, central diabetes insipidus; Chronic headache; Progressive visual deterioration	Level III	High Quality
Muthusamy et al.	2019	India	Case Report	1	52	F	Suprasellar	Worsening headache, recurrent giddiness	Level V	Fair Quality
Suetens et al.	2019	Belgium	Case Report	1	43	M	Third Ventricle	Increasing visual disturbances, bilateral hemianopsia	Level V	High Quality
Chen et al.	2020	China	Case Report	1	33	F	Third Ventricle	Progressive obesity, menstrual disturbance (hypomenorrhea, amenorrhea)	Level V	High Quality
Cui et al.	2020	China	Case Report	1	51	M	Third Ventricle (Anterior)	Frontal headache	Level V	High Quality
Yang et al.	2020	China	Case Series	4	41, 27, 67, 35	2 M, 2 F	Third Ventricle	Dizziness, fatigue, diabetes insipidus, electrolyte disturbance; Visual deterioration, somnolence; Dizziness	Level IV	High Quality
Yao et al.	2020	China	Retrospective Cohort	16	18, 25, 32, 33, 38, 41, 43, 44, 46, 47, 52, 53, 53, 53, 59, 30	7 M, 9 F	Suprasellar & Third Ventricle (x13)Third Ventricle (x3)	Intermittent headache; Vomiting; Light-headedness; Vision loss; Sleepiness; Polyuria, polydipsia	Level III	Fair Quality
Zhang et al.	2020	China	Retrospective Cohort	12	27, 35, 41, 67, 66, 32, 50, 30, 43, 43, 44, 53	5 M, 7 F	Third Ventricle	Visual deterioration, somnolence, sexual dysfunction; Menstrual disorder, fatigue, irritability, memory deterioration; Headache, ataxia; Diabetic insipidus; Shuffle gait, incontinence; Epileptic seizure	Level III	High Quality
Chen et al.	2021	China	Case Report	1	51	M	Third Ventricle	Recurrent head pain, intermittent limb weakness	Level V	High Quality
Dias et al.	2021	Portugal	Case Report	1	57	F	Suprasellar & Third Ventricle	Recent unusual headaches, facial paresthesia	Level V	High Quality
Hung et al.	2021	Vietnam	Case Report	1	45	F	Third Ventricle (Anterior)	Headache, memory deficits, visual disturbances, weight gain	Level V	High Quality
Scholl et al.	2021	USA	Case Report	1	48	M	Third Ventricle	Acutely worsening ataxia, forgetfulness, nausea/vomiting, headache, blurred vision	Level V	High Quality
Yang et al.	2021	China	Case Report	1	50	F	Third Ventricle	Cognitive dysfunction, disorientation, declined visual acuity	Level V	High Quality
Zhang et al.	2021	China	Case Report	1	53	F	Third Ventricle (Anterior)	Fever from aspiration pneumonia (History: bedridden, deteriorating consciousness, drowsiness)	Level V	High Quality
Konovalov et al.	2023	Russia	Case Series	10	27, 14, 65, 60, 12, 44, 26, 31, 36, 31	5 M, 5 F	Third Ventricle (Posterior/General)	Headache; Diabetes insipidus; Impaired memory and vision; Asymptomatic; Nausea, vomiting; Mental disorders; Drowsiness, amenorrhea; Weight loss	Level IV	High Quality
Mohin et al.	2023	India	Case Report	1	21	M	Third Ventricle	Headache, vomiting, memory impairment	Level V	Fair Quality
Oda et al.	2023	Japan	Case Report	1	44	M	Third Ventricle	Initial occipital headache. Later: short memory disorder, daytime sleepiness, hydrocephalus	Level V	High Quality
Huang et al.	2024	China	Retrospective Cohort	13	Mean 35.1 (7–51)	7 M, 6 F	Suprasellar & Third Ventricle	Intermittent dizziness and headache, some with vomiting, visual field deficits, memory loss (Aggregate)	Level III	High Quality
Feng et al.	2025	China	Case Series	5	31, 45, 32, 44, 49	1 M, 4 F	Suprasellar & Third Ventricle	Headache; Visual deficits; Incidental finding	Level IV	High Quality

F: Female; GI: Gastrointestinal; M: Male; mo: months; SIADH: Syndrome of Inappropriate Antidiuretic Hormone Secretion; UK: United Kingdom; USA: United States of America; V3: Third Ventricle; yrs: years

### Quality, risk of bias, and level of evidence assessment

Critical appraisal of the 94 studies revealed a predominance of lower-level evidence typical of rare pathologies, yet individual reporting quality remained largely favorable (Table 1). Using AANS/CNS grading criteria, only five studies met Level III evidence criteria, including the high-quality cohorts by Bielle et al. [6], Johannes et al. [88], Zhang et al. [94], and Huang et al. [95], alongside the fair-quality cohort by Yao et al. [93]. Twelve studies were designated as Level IV case series, of which the majority were rated as high quality [1, 8, 21, 46, 82, 92, 96, 97], while three studies were assessed as fair quality [3, 20, 60]. Notably, one series was deemed low quality due to insufficient data [75]. The remaining 70 publications were classified as Level V case reports. Despite inherent selection and publication bias associated with Level V evidence, overall reporting quality was high, enabling robust pooled data extraction [4, 7, 10, 12, 22–36, 38–45, 47–59, 61, 63–67, 69–74, 76–87, 90, 91, 98–104]. Only a small subset of case reports were classified as fair quality [37, 43, 62, 68, 86, 89, 105] (Supplementary Tables S2–S4).

### Clinical presentation and symptomatology

A pooled analysis of presenting symptoms was performed on 160 patients, excluding 38 cases from larger cohorts where individual symptom data were not specified (Table 2). The most frequent symptom was headache (51.3%), typically attributed to obstructive hydrocephalus or elevated intracranial pressure. Visual disturbances affected 37.5%, manifesting as decreased acuity, diplopia, and visual field defects: bitemporal hemianopsia, consistent with suprasellar extension and chiasmatic compression. Cognitive and behavioral disturbances affected 24.4%, presenting with short-term memory loss, confusion or personality changes.

**Table 2:** Pooled Analysis of Presenting Symptoms

Symptom Category	Frequency (*n*)	Percentage (%)	Clinical Notes
Headache	82	51.3%	The most common presentation, often due to obstructive hydrocephalus or elevated intracranial pressure.
Visual Disturbances	60	37.5%	Includes decreased acuity, bitemporal hemianopsia, and diplopia. Caused by compression of the optic chiasm (suprasellar extension).
Cognitive & Mental Status	39	24.4%	Frequently described as short-term memory loss (amnesia), confusion, dementia-like progression, or personality changes.
Sleep/Somnolence	31	19.4%	Ranges from “daytime sleepiness” and lethargy to “drop attacks” (syncope) and stupor. Likely related to hypothalamic compression.
Endocrine/Hormonal	29	18.1%	Includes Diabetes Insipidus (polyuria/polydipsia), amenorrhea, weight gain (hyperphagia), and hypothyroidism.
Nausea/Vomiting	22	13.8%	Typically accompanies headache as a sign of acute hydrocephalus.
Motor/Ataxia	17	10.6%	Gait disturbance, ataxia, or hemiparesis. Often seen in large tumors compressing the midbrain or thalamus.
Seizures	6	3.8%	A rare presentation [42, 76].
Incidental/Asymptomatic	3	1.9%	Rare: most tumors are symptomatic at diagnosis.
Other symptoms	30	19%	Dizziness/giddiness: 14 cases [27, 33, 42, 60, 81, 89, 92, 94, 99, 104]. Syncope/LOC: 6 cases [1, 8, 30, 46, 47, 66]. Loss of smell: 2 cases [7, 12]. Fever: 2 cases [1, 43, 100]. GI symptoms: 2 cases [1, 12]. Hypertension: 1 case [8]. SIADH/electrolyte: 3 cases[72, 84, 92].

*Percentages sum to > 100% as most patients had multiple symptoms*

Beyond this classic triad of headache, visual deficits, and cognitive decline, symptoms related to hypothalamic dysfunction were prominent. Sleep disturbances, ranging from lethargy and daytime somnolence to “drop attacks” and stupor, were noted in 15.7% of patients. Endocrine dysfunction was present in 14.6%, presenting as diabetes insipidus, amenorrhea, weight gain (hyperphagia), or hypothyroidism. Nausea and vomiting (13.8%) frequently accompanied headaches, while motor deficits (gait disturbances, ataxia) were observed in 10.6%, likely due to midbrain or thalamic compression of the structures. A minority of patients presented with atypical or rare symptoms, with dizziness or giddiness for 7%, and syncope or Loss of Consciousness (LOC) the primary presenting feature for 3%. Rare sensory and systemic findings included loss of olfactory sensation, fever, gastrointestinal symptoms, hypertension, and Syndrome of Inappropriate Antidiuretic Hormone (SIADH) or electrolyte disturbances (Table 2).

### Radiological features

The radiological appearance of chordoid gliomas are characteristically distinct, though specific signal variations exist that may correlate with emerging molecular phenotypes. In a pooled analysis of 191 patients, hydrocephalus was the most frequent associated finding, reported in 20% of cases, reflecting the tumor’s propensity to obstruct the ventricular system. Cystic changes were identified in 17.5%, while calcification was less common, noted in 7.5% of cases.

Magnetic Resonance Imaging (MRI) analysis of 84 patients with reported T1-weighted data revealed that 59.5% of tumors appeared isointense, a classic presentation [1, 81]. However, 31.0% were hypointense, a finding more prevalent than historically recognized and well-represented in recent large cohorts [60, 95]. Rare variations included heterogeneous signals (3.6%) associated with complex cystic or hemorrhagic components [23], and hyperintensity (2.4%), which were explicitly reported in only two cases [96].

T2-weighted and FLAIR imaging were reported in 45% of patients, demonstrating a bimodal signal pattern that may have biological implications. While classical hyperintense appearances were observed in 51.7%, a distinct isointense phenotype was identified in 34.8% of patients. Isointense patterns have been increasingly documented in recent studies [95, 97], and associated with the BRAF V600E mutation. Heterogeneous T2 signals were observed in 11.2% of cases, typically corresponding to large, cystic, or hemorrhagic lesions [64, 65].

Contrast enhancement imaging (performed in 73% of patients) typically demonstrated substantial homogeneous enhancement (81.7%). Heterogeneous enhancement was noted in 16.2%, often in tumors with cystic degeneration [70, 89, 95]. Atypical non-enhancing or poorly enhancing variants were exceptionally rare (2.1%) [101, 105]. Diffusion-Weighted Imaging (DWI) findings, though limited to only 12 reported cases, supported the low-grade nature of these neoplasms, with 91.7% showing no restriction [95, 99, 101], with restricted diffusion observed in only a single hypercellular case [100].

### Tumor location and extent

Anatomical analysis of 192 patients confirmed that chordoid gliomas were predominantly centered in the anterior third ventricle and hypothalamic regions. The most frequent localization pattern was suprasellar extension, observed in 44.3%, with these tumors typically originating in the third ventricle but extended inferiorly to fill the suprasellar cistern, often resulting in significant optic chiasm and hypothalamus compression. In 40.6% of cases, tumors were described as confined strictly to the third ventricle, occupying the anterior, middle, or posterior floor, without explicit mention of suprasellar extension. A smaller tumor subset (2.6%) was localized to the foramen of Monro or lamina terminalis. Notably, ectopic or atypical presentations were identified in 11.5% with these rare cases involving tumors extending into or originating from structures outside the standard ventricular boundaries, including the thalamus [50], corona radiata [43], and midbrain, highlighting the potential for atypical anatomical spread.

### Surgical approaches and treatments

**Fig. 2:**
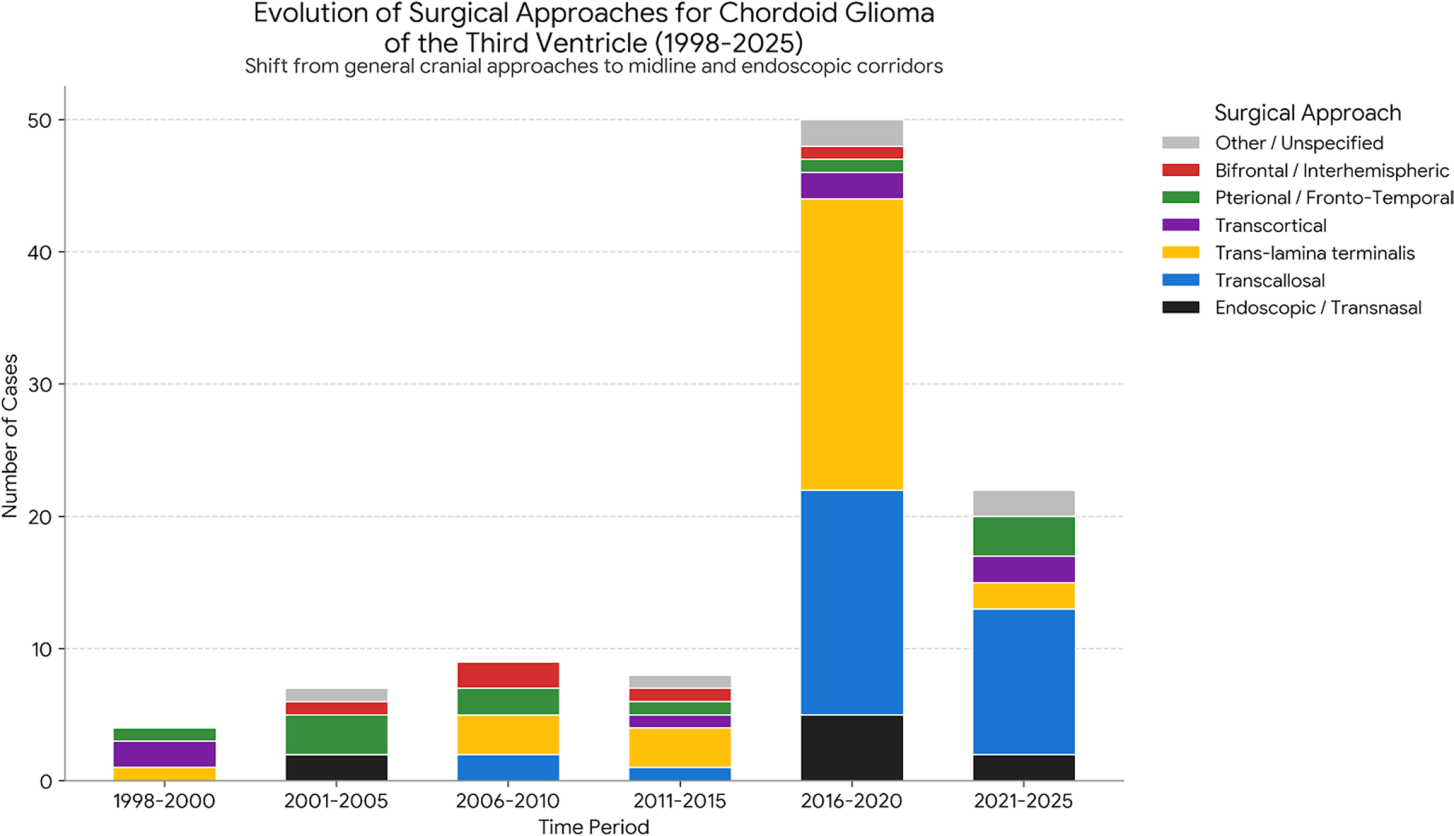
Evolution of Surgical Approaches for Chordoid Gliomas of the Third Ventricle (1998–2025). From 1998–2010, management was characterized by a variety of standard cranial approaches, with frequent use of Pterional/Fronto-Temporal and Transcortical routes. From 2016–2025, management shifted toward midline approaches. The Transcallosal and Trans-lamina terminalis corridors became the dominant strategies, accounting for the vast majority of cases from 2016–2025. This aligns with the increasing recognition of these routes as the safest corridors for preserving hypothalamic function in third ventricle surgery. Endoscopic (transnasal or intraventricular) approaches emerged between 2020–2025, primarily for biopsies or specific anatomical configurations, though they remain less common than open microsurgery

An analysis of the 100 cases with detailed operative descriptions indicated that surgical strategies were heavily influenced by the tumor’s relationship with the third ventricle and the hypothalamus (Fig. 2). The interhemispheric transcallosal approach was the most frequently utilized corridor, employed in 31 cases. The trans-lamina terminalis approach (including fronto-basal interhemispheric variations) was the second most common technique, used in 28 cases to access the anterior third ventricle. Standard pterional or fronto-temporal craniotomies were used in 11 cases, while the transcortical transventricular approach was documented in 10 cases, providing direct access through the lateral ventricle. Less common strategies included endoscopic endonasal approaches (9 cases), primarily for biopsy or specific anatomical presentations, and general bifrontal or subfrontal craniotomies utilized for complex or unspecified scenarios.

**Fig. 3:**
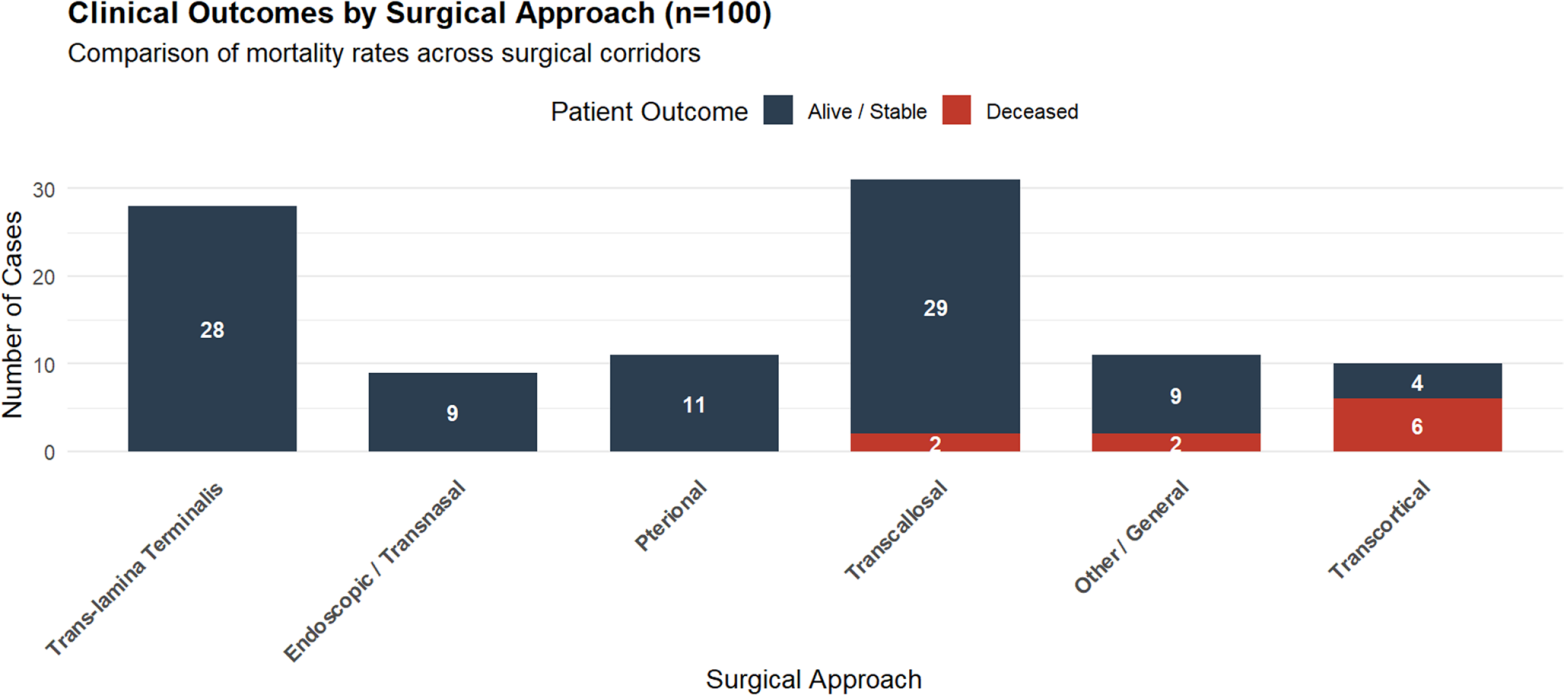
Clinical Outcomes Stratified by Surgical Approach (100 patients). Pooled analysis of 100 cases comparing mortality and survival for the five major surgical corridors. Black bars represent patients who are alive or stable at last follow-up. Red bars represent disease-related mortality (perioperative death or progression). Key Findings: The Trans-lamina terminalis (*n* = 28) and Endoscopic/Transnasal (*n* = 9) approaches demonstrated 100% survival in this cohort. In contrast, historical Transcortical approaches (*n* = 10) were associated with significantly higher mortality (60%), primarily due to pulmonary embolism and respiratory complications in the pre-modern era. The Transcallosal approach (*n* = 31) remains the most utilized corridor with a high safety profile in modern series

A descriptive analysis of reported mortality outcomes across these surgical corridors showed that no mortality events were recorded within the small endoscopic subgroup (9 cases), whereas historical transcortical approaches (10 cases) demonstrated higher descriptive mortality rates (Fig. 3).

Surgical resection remains the main management for chordoid gliomas, utilized in approximately 88% of this patient population. GTR was achieved in 56%, reflecting a trend toward more aggressive removal in recent years, while STR was performed in 32%, often intentionally to preserve critical hypothalamic and optic structures adhering to the tumor capsule. Biopsy alone was reserved for 10% of patients, typically via stereotactic or endoscopic means when the lesion was deemed unresectable or for diagnostic confirmation prior to observation-only. Adjuvant therapy was uncommon; radiotherapy was administered in only 8%, primarily for residual or recurrent disease. Reported modalities included GKRS, conventional radiotherapy, and rare instances of brachytherapy or craniospinal irradiation. Chemotherapy was extremely rare (< 1%), with only one reported case of temozolomide use for disseminated disease [74]. A purely observational strategy was adopted in only 1.5%, for asymptomatic or stable patients.

### Survival outcomes

**Fig. 4:**
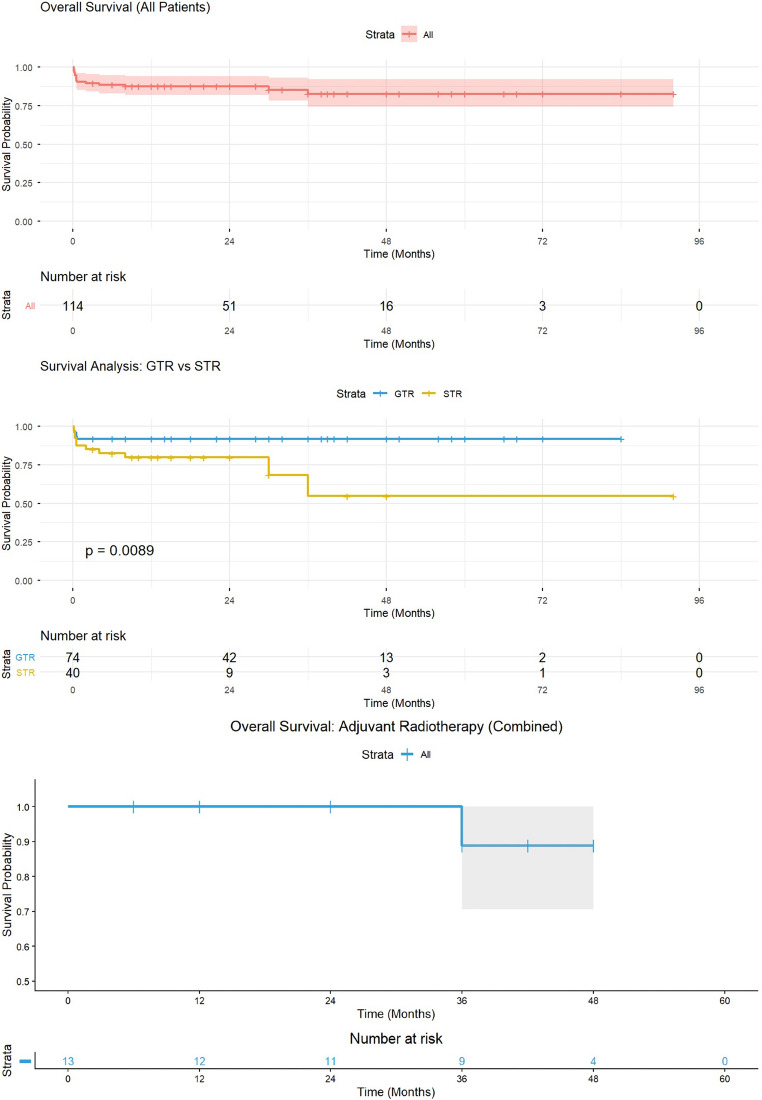
Overall Survival (All Patients). Overall Survival: Top panel displays survival probability for the entire patient cohort (*n* = 114) over 96-months. Shaded red represents the 95% confidence interval. Survival Analysis: Middle panel compares survival outcomes for Gross Total Resection (GTR, blue line, *n* = 74) versus Subtotal Resection (STR, yellow line, *n* = 40). GTR demonstrated a statistically significant survival advantage compared to STR (log-rank *p* = 0.0089). Overall Survival with Adjuvant Radiotherapy: Bottom panel shows survival probability for patient subgroup receiving adjuvant radiotherapy (*n* = 13). Shading from 36 to 48 months shows 95% confidence interval

Kaplan-Meier survival analysis assessed the long-term prognosis of the entire patient cohort (Fig. 4), with the overall survival probability of 87.6% at 1 year (95% CI: 0.817–0.939). Long-term follow-up demonstrated a stabilization of mortality risk, with survival probabilities plateauing at 82.6% at both 3- and 5-year intervals (95% CI: 0.742–0.920).

A comparative survival analysis stratified by extent of resection revealed statistically significant improved survival for GTR (Fig. 4). GTR demonstrated a superior 1-year survival rate of 91.9% (95% CI: 0.859–0.983), which remained stable at 91.9% through the 3-year and 5-year intervals, indicating no additional mortality events in this cohort after the first year. In contrast, the STR group exhibited a progressive survival decline of 79.8% at 1-year (95% CI: 0.682–0.933) and 54.7% at 3- and 5-years (95% CI: 0.314–0.953). Log-Rank testing confirmed that GTR was associated with significantly better overall survival compared to STR (*p* = 0.0089).

Finally, pooled survival analysis for patients who received radiotherapy demonstrated acceptable long-term survival: The overall survival rate of 92.3% remained stable through the 5-year benchmark. While the majority of patients achieved durable local control with no evidence of progression, only one single disease-related mortality was recorded at 36-months (Fig. 4).

### Genetics and molecular pathogenesis

Molecular profiling has recently established chordoid gliomas as genetically distinct neoplasms defined by specific recurrent driver mutations. The hallmark alteration is the PRKCA D463H mutation, identified in 87.5–100% of cases in recent major cohorts. A smaller, mutually exclusive subgroup harbors the BRAF V600E mutation (12.5%), which appears to correlate with specific radiological phenotypes. Cytogenetic studies have further characterized the tumor landscape by identifying recurrent copy-number variations, particularly deletions at 9p21 (CDKN2A) and 11q13 (MEN1), while consistently confirming the absence of canonical diffuse glioma markers, including IDH1/2 and TP53 mutations. Table 3 provides a detailed summary of included genetic studies and their findings.

**Table 3:** Genetic Profiling and Molecular Characteristics of Chordoid Gliomas in the Included Studies

Study	Method of Analysis	Key Positive Genetic Findings (Mutations/CNVs)	Key Negative Findings (Absent Alterations)	Notes
Reifenberger et al. (1999)	CGH, SSCP, Differential PCR	**None.** No chromosomal imbalances or amplifications were detected in this series.	• No *TP53* mutations • No *CDKN2A* mutations/deletions • No amplification of *EGFR*, *CDK4*, *MDM2*	Early study suggesting these tumors are genetically distinct from diffuse astrocytomas.
Horbinski et al. (2009)	Array CGH, FISH, Sequencing	• **Losses at 9p21** (*CDKN2A* locus) in 5/5 cases • **Losses at 11q13** (*MEN1* locus) in 5/5 cases • **Monosomy of Chrs 9 and 11** (approx. 20–50% of cells)	• No *TP53* mutations • No *EGFR* amplification	Suggested potential tumor suppressor roles for *p16* (9p21) and *menin* (11q13) in tumorigenesis.
Bielle et al. (2015)	Sanger Sequencing	**TTF-1 (NKX2-1) Expression**: Confirmed as a consistent marker (though protein-level, it reflects gene expression).	• No *IDH1* (R132) mutations • No *IDH2* (R172) mutations • No *BRAF* V600E mutations	Confirmed genetic distinction from low-grade infiltrating gliomas and *BRAF*-mutated tumors.
Erwood et al. (2017)	Cytogenomic Microarray	**Extensive Copy Number Abnormalities**: • **Gains**: Chr 8 (whole), 1q, 3p, 7q • **Losses**: Chr 9 (whole, incl. *CDKN2A*), Chr X, 6q, 7q	N/A	Case of rapid progression. The genomic instability (e.g., loss of *CDKN2A*) may correlate with the aggressive behavior.
Yao et al. (2020)	Sanger Sequencing, NGS, ARMS-PCR	• **PRKCA D463H mutation**: Found in **14 of 16** cases (87.5%). • **BRAF V600E mutation**: Found in **2 of 16** cases (12.5%)	• Negative for *IDH1* R132H (IHC).	*BRAF* V600E was exclusively found in cases with atypical **histiocyte-like features**, suggesting a potential new histological subtype.
Zhang et al. (2021)	Whole-Exome Sequencing (WES)	• **PRKCA D463H mutation** (c.1387G > C): Identified as a specific driver. • **25 Shared Mutations** with concurrent meningioma (e.g., *CGREF1*, *CDC27*, *TTN*).	• No *IDH1/2* mutations • No *TP53*, *ATRX*, *TERT* mutations	First report of *PRKCA* D463H in a patient with concurrent meningioma. Confirmed *PRKCA* as a hallmark.
Feng et al. (2025)	Sanger Sequencing	• **PRKCA D463H mutation**: Found in **100% (5/5)** of cases. • **SOX2 Expression**: Diffuse/Strong in all cases (genetic marker of glial origin)^21^.	• Negative for *BRAF* V600E • Negative for *IDH* mutations (implied by glioma comparison)	Reaffirmed *PRKCA* D463H as the defining genetic characteristic of Chordoid Glioma.

## Discussion

Chordoid gliomas of the third ventricle represent a rare neurosurgical challenge. Since their description by Brat et al. in 1998 [1], nearly 200 cases have been reported, making them among the rarest adult primary brain tumors [2, 106]. Despite their WHO Grade II and histologically low-grade classification, they are associated with substantial morbidity and mortality and often behave more aggressively than expected [13, 44]. This systematic review of 198 patients from 94 studies represents the largest analysis to date and addresses key gaps in the management of this rare tumor. The anterior third ventricular location with close involvement of the hypothalamus, optic apparatus, and major vessels creates major neurosurgical challenges which distinguish these from low-grade gliomas in other brain regions. Unlike diffuse gliomas that infiltrate the brain parenchyma, chordoid gliomas are well-circumscribed masses that should, in theory, be amenable to GTR. However, their dense adhesion to the hypothalamus, optic apparatus and the peri-third ventricular region may make aggressive resection undesirable due to a risk of devastating neurological and endocrine complications. This fundamental tension between oncological principles favoring GTR and the imperative to preserve QoL by protecting critical neural structures has made management decisions particularly complex and led to variable treatment approaches [69]. Indeed, perioperative mortality has been reported to reach as high as 32% in early surgical series, primarily due to hypothalamic injury [13], underscoring the risk with aggressive resection.

Previous literature on chordoid gliomas has been limited primarily to case reports and small case series, with only one systematic review and some case series with literature reviews that have attempted to synthesize the available evidence. However, the sole comprehensive prior systematic literature review by Ampie et al. (2015) [13], did not incorporate the molecular profiling that is revolutionizing brain tumor classification and management. The need for an updated, comprehensive analysis incorporating modern molecular characterization, contemporary surgical techniques, and long-term survival data is paramount to guide evidence-based clinical decision-making for this rare but clinically significant tumor.

Our pooled analysis revealed a characteristic clinical presentation dominated by the classic triad of headache, visual disturbances, and cognitive dysfunction. This symptom constellation reflects the tumor’s location at critical neuroanatomical structures. The high prevalence of headache is primarily attributable to obstructive hydrocephalus from third ventricular mass effect. Visual symptoms (decreased acuity, diplopia, bitemporal hemianopsia) result from suprasellar extension and optic chiasm compression. The significant burden of hypothalamic dysfunction documented herein underscores the tumor’s predilection for the hypothalamic region. Given the high risk of postoperative endocrine complications, early involvement of neuroendocrinology is recommended to guide perioperative hormone management and postoperative replacement strategies, particularly in patients undergoing STR or presenting with pre-existing dysfunction.

Recognition of hypothalamic dysfunction has important implications for surgical planning and patient counseling. Preoperative hypothalamic symptoms often predict postoperative morbidity because tumor adherence to hypothalamic structures increases surgical risk [13], and these deficits may persist or worsen after surgery, contributing to long-term disability even after GTR. Consequently, preserving hypothalamic function remains a central challenge for chordoid glioma surgery. Intraoperative strategies such as minimal hypothalamic dissection, sharp dissection near the tuber cinereum, and neuronavigation with MR-tractography may reduce injury to periventricular nuclei. For predominantly anterior midline tumors, the trans-lamina terminalis approach may also provide direct access with reduced hypothalamic manipulation [88, 95].

Molecular profiling has revolutionized our understanding of chordoid gliomas. The recurrent PRKCA D463H mutation is now established as a robust diagnostic hallmark, driving oncogenesis through distinct pathways separate from the IDH-mutant mechanisms of diffuse gliomas [107–109]. A smaller subset of tumors harboring the BRAF V600E mutation has also been identified, displaying a genotype-phenotype correlation with MRI T2 isointensity [81, 95]. While this molecular distinctiveness possesses clear, established relevance for differential diagnosis and pathological categorization, its therapeutic translation remains entirely theoretical. These biological alterations highlight speculative pathways for potential precision therapies in the future [9], but clinical evidence supporting targeted treatments for this pathology is currently non-existent. Collectively, these markers confirm that chordoid gliomas represent a unique biological entity, though these genetic findings must currently be regarded as strictly hypothesis-generating rather than clinically directive for active medical management.

Our survival analysis demonstrates a significant statistical association between GTR and improved overall survival compared to STR, consistent with previous retrospective reports [6, 13]. This observed survival difference may reflect the oncological benefit of complete excision facilitated by the tumor’s well-defined borders [1, 9], though this finding remains highly vulnerable to confounding by patient selection and baseline tumor characteristics. However, aggressive resection must be balanced against surgical risk, as perioperative mortality rates up to 32% have been reported due to hypothalamic injury [44]. Crucially, because long-term functional data are insufficient in the literature to reliably weigh surgical morbidity against survival extensions, the choice of strategy must center on preserving the patient’s baseline neurological status rather than pursuing radical radiographic clearance. Consequently, tumors with dense adherence to the tuber cinereum or infundibulum may require prioritizing functional preservation rather than radical resection.

**Fig. 5:**
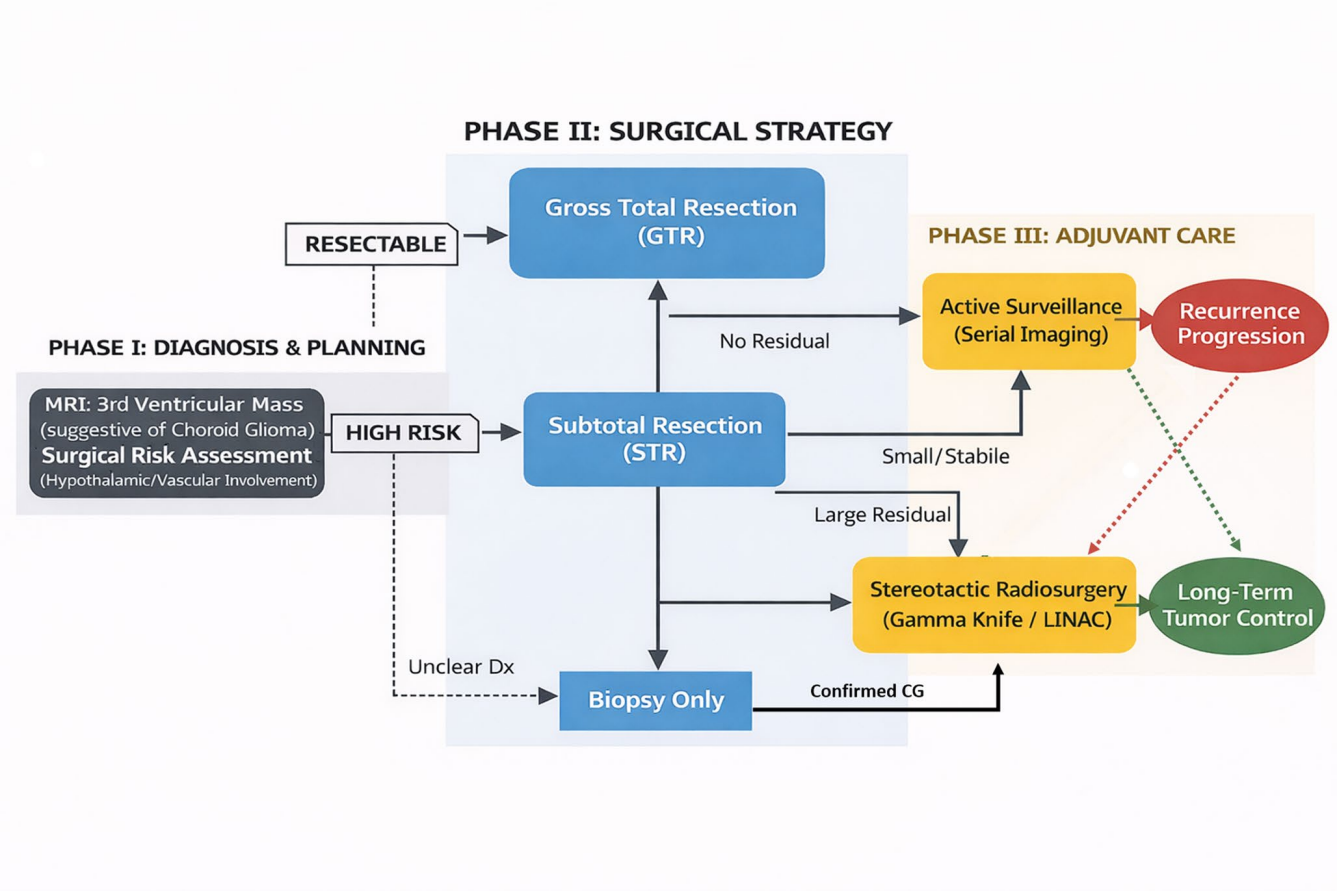
Proposed Clinical Management Algorithm for Chordoid Gliomas. This phased clinical pathway diagram was developed from pooled analysis of 198 cases. Management is stratified into three distinct phases to optimize functional outcomes and tumor control. Phase I (Diagnosis & Planning, Grey shading): Begins with radiological identification of a third ventricular mass. A critical risk assessment to determine surgical candidacy is evaluating hypothalamic adherence and vascular involvement. Phase II (Surgical Strategy, Blue shading): Gross Total Resection (GTR) is the preferred goal for resectable lesions. Subtotal Resection (STR) or Biopsy is indicated when the tumor is adherent to critical structures (high-risk anatomy) to spare neurological function. Phase III (Adjuvant Care, Yellow shading): Patients with large residual tumor or confirmed biopsy diagnoses proceed directly to Stereotactic Radiosurgery (SRS) or Gamma Knife. Small, stable residuals are managed via Active Surveillance. Outcomes: Green ovals denote long-term tumor control. The red dotted line represents the salvage pathway, where recurrence or progression during surveillance triggers rescue radiotherapy

Management should therefore be individualized. While GTR is frequently attempted when anatomically favorable planned STR with adjuvant therapy represents a crucial alternative for high-risk lesions where adjacent structures are jeopardized, with radiosurgery such as GKRS reported as a potential option for residual disease despite limited evidence [14, 110, 111]. Fractionated stereotactic radiotherapy has also been utilized for select cases; however, its efficacy and long-term toxicity profile remain poorly defined [4, 10, 43]. Conversely, in young patients with indolent residuals, a “watch-and-wait” strategy may be preferred to defer the long-term cognitive and endocrine sequelae of chemotherapy and radiation [112–114]. Based on the synthesis of these reported survival trends, surgical corridors, and risk factors, we propose a phased management algorithm (Fig. 5) to serve as a useful educational framework and an expert-informed, hypothesis-generating conceptual tool during the decision-making process. This framework considers GTR when anatomically favorable based on reported case patterns, while STR followed by observation or adjuvant radiotherapy remains a reasonable strategy for high-risk, densely adherent lesions. This structured pathway integrates reported surgical and adjuvant data to assist clinicians in balancing long-term tumor control against the preservation of vital hypothalamic function.

A key finding of this review is the paucity of postoperative QoL data. Existing literature, largely case reports and small series, emphasizes extent of resection and recurrence-free survival while often overlooking long-term functional outcomes. Given the tumor’s close relationship with critical diencephalic structures, particularly the hypothalamus and fornices, aggressive resection carries risks of permanent endocrine dysfunction, memory impairment, and neuropsychological deficits. Thus, radiographic clearance alone may not represent true clinical success. Future studies should incorporate standardized functional outcome measures (e.g., 36-Item Short Form Survey (SF-36) and long-term Karnofsky Performance Status) to determine whether the benefits of radical resection outweigh its functional costs.

Our study presents a critical point of divergence from historical literature regarding surgical corridors, morbidity, and clinical predictors. In 2015, Ampie et al. [13] evaluated patients and concluded that postoperative complications did not correlate with tumor size, age, or extent of resection, while highlighting a lower complication trend for the trans-lamina terminalis approach. However, that analysis carried clear methodological limitations by modern meta-analytic standards; it pooled scattered case reports into a single cohort and ran univariate tests without assessing inter-study heterogeneity via metrics like the I^2 statistic. This approach is highly vulnerable to publication bias, which frequently underreports negative outcomes and can mask true clinical risks. In contrast, the current study’s expanded dataset captures a distinct modern pivot toward the interhemispheric transcallosal corridor. This transition is heavily driven by contemporary technological adjuncts, including neuronavigation, tractography, and endoscope-assisted microsurgery, which mitigate traditional risks of callosal and forniceal injury while offering superior top-down visualization for large lesions filling the middle and posterior third ventricle. This selection of surgical corridor is inextricably linked to the prognostic realities of the disease. Our survival analytics demonstrate that while GTR is associated with superior long-term survival rates in the literature compared to STR, its mortality risk is heavily concentrated within the immediate postoperative year. This pattern reflects the acute surgical dangers of aggressive dissection near critical diencephalic structures and shows that hypothalamic dysfunction occurs across both groups regardless of tumor size. Utilizing advanced corridors paired with modern visualization may help minimize early perioperative morbidity for teams attempting complete resections, which are linked to durable long-term tumor control in retrospective series.

Finally, while our descriptive results noted varying mortality rates among different corridors, such as a lack of mortality events in the emerging endoscopic endonasal subgroup, these findings cannot be interpreted as a measure of comparative effectiveness. These subgroup outcomes are heavily confounded by selection bias and treatment era. Endoscopic approaches are heavily selected for contemporary cases with favorable, non-adherent tumor anatomy or are restricted to safe diagnostic biopsies. Conversely, the higher mortality frequencies observed in historical transcortical-transventricular routes reflect an older neurosurgical era operating prior to the routine integration of micro-endoscopic visualization and modern neuro-intensive care. Due to these overlapping biases and the small sizes of the pooled subgroups, a definitive clinical superiority of one corridor over another cannot be established, and the selection of the surgical route must remain strictly individualized based on the specific three-dimensional topography of the tumor.

### Strengths and limitations

Representing the most comprehensive analysis of chordoid glioma to date, this systematic review synthesizes data from 198 patients across 94 studies, utilizing robust quality assessment tools and Kaplan-Meier survival analysis to provide quantitative evidence. In addition to evaluating surgical outcomes, this study uniquely incorporates contemporary molecular profiling, particularly PRKCA and BRAF mutations, alongside detailed radiological characterization to inform clinical decision-making.

These strengths, however, are counterbalanced by limitations inherent to the study of rare pathologies, including publication bias favoring positive or successful outcomes, significant heterogeneity in data reporting standards, and the absence of randomized controlled trials. Most critically, the interpretation of our findings is constrained by a profound, literature-wide reporting gap regarding long-term functional and QoL outcomes. Because historical literature almost exclusively highlights the extent of resection and recurrence-free survival while omitting standardized functional metrics (such as the SF-36 or long-term Karnofsky Performance Status), it is impossible to reliably determine whether the survival benefits of radical resection outweigh its severe functional costs. This widespread omission severely limits the ability to make definitive clinical recommendations regarding aggressive surgical clearance.

Furthermore, while the statistical advantage of aggressive resection is evident in the pooled data, these findings remain highly vulnerable to selection bias, incomplete longitudinal follow-up, and the scarcity of molecular details in historical case reports. The lack of individual-level patient data also precluded multivariable adjustment for confounders, and survival analyses based on pooled case reports and small case series cannot fully account for biological heterogeneity or treatment variability, thereby limiting causal inference.

## Conclusions

This systematic review demonstrates a significant statistical association between GTR and superior 5-year survival rates in reported cases. However, because functional morbidity and long-term quality-of-life data are profoundly underrepresented throughout the literature, these radiographic survival advantages must be interpreted with extreme caution. This statistical benefit cannot support a direct causal inference, as patients undergoing STR likely possessed higher-risk, more adherent lesions. Consequently, surgical decision-making demands a nuanced balance between achieving tumor control and protecting critical diencephalic structures. Because the true functional cost of aggressive resection remains largely unquantified in historical series, functional preservation must remain the primary clinical priority over radical radiographic clearance.

The identification of the hallmark PRKCA D463H mutation represents a pivotal advancement in the biological understanding and diagnostic accuracy of chordoid gliomas, reinforcing their distinction from other low-grade gliomas. While this insight provides a strong framework for future investigative research, its therapeutic utility remains unproven. In the long term, these markers may lay a conceptual foundation for future precision-based strategies, where experimental multimodal management or targeted therapies could eventually be explored. However, until such targeted options emerge, clinicians must rely on tailored surgical strategies that aggressively safeguard the patient’s baseline neurological and endocrine status rather than pursuing high-risk complete resections.

## Supplementary Information

Below is the link to the electronic supplementary material.

Supplementary Material 1 (DOCX 52.3 KB) 

## Data Availability

The datasets generated and/or analyzed for this study are available from the corresponding author on reasonable request.
