# Supplementary material for: Surgical strategies and long-term survival for third ventricle chordoid gliomas: a systematic review and clinical algorithm
Source: Neurosurg Rev. 2026 Jun 25;49(1):456. doi: 10.1007/s10143-026-04375-x (PMC13294298; doi:10.1007/s10143-026-04375-x)
Supplement: Supplementary file 1 — Supplementary Material 1 (DOCX 52.3 KB) [file 10143_2026_4375_MOESM1_ESM.docx]

**Table S1. Table S1. Detailed Search Strategy and Database Yields for Chordoid Gliomas of the Third Ventricle**

| **Surgical Strategies and Long-Term Survival for Third Ventricle Chordoid Gliomas: A Systematic Review and Clinical Algorithm**  Medline (through PubMed), Scopus, Embase, and Web of Science online databases were searched from inception through October 21, 2025*.* | | |
| --- | --- | --- |
| *Terms:*  *#1= ('chordoid glioma' OR 'chordoid glioma of the third ventricle' OR 'chordoid tumor' OR 'chordoid neoplasm' OR 'suprasellar chordoid glioma' OR 'anterior third ventricle glioma' OR 'hypothalamic chordoid glioma' OR 'diencephalic chordoid glioma' OR 'chordoid glioma brain tumor' OR 'chordoid glioma CNS' OR 'chordoid glioma of the central nervous system')*  *#2= ('third ventricle' OR 'third ventricles' OR '3rd ventricle' OR '3rd ventricles' OR 'anterior third ventricle' OR 'anterior third ventricles' OR 'suprasellar region' OR 'suprasellar space' OR hypothalamus OR hypothalamic OR diencephalon OR diencephalic OR 'interventricular foramen' OR 'foramen of Monro')*  *#3=1 AND 2* | | |
| ***Database*** | ***Search Terms*** | ***Number of articles*** |
| *Scopus* | *(TITLE-ABS-KEY("chordoid glioma" OR "chordoid glioma of the third ventricle" OR "chordoid tumor" OR "chordoid neoplasm" OR "suprasellar chordoid glioma" OR "anterior third ventricle glioma" OR "hypothalamic chordoid glioma" OR "diencephalic chordoid glioma" OR "chordoid glioma brain tumor" OR "chordoid glioma CNS" OR "chordoid glioma of the central nervous system")*  *AND*  *TITLE-ABS-KEY("third ventricle" OR "third ventricles" OR "3rd ventricle" OR "3rd ventricles" OR "anterior third ventricle" OR "anterior third ventricles" OR "suprasellar region" OR "suprasellar space" OR hypothalamus OR hypothalamic OR diencephalon OR diencephalic OR "interventricular foramen" OR "foramen of Monro")* | *136* |
| *Web of Science* | *TS=("chordoid glioma" OR "chordoid glioma of the third ventricle" OR "chordoid tumor" OR "chordoid neoplasm" OR "suprasellar chordoid glioma" OR "anterior third ventricle glioma" OR "hypothalamic chordoid glioma" OR "diencephalic chordoid glioma" OR "chordoid glioma brain tumor" OR "chordoid glioma CNS" OR "chordoid glioma of the central nervous system")*  *AND*  *TS=("third ventricle" OR "third ventricles" OR "3rd ventricle" OR "3rd ventricles" OR "anterior third ventricle" OR "anterior third ventricles" OR "suprasellar region" OR "suprasellar space" OR hypothalamus OR hypothalamic OR diencephalon OR diencephalic OR "interventricular foramen" OR "foramen of Monro"))* | *154* |
| *PubMed* | *(("chordoid glioma"[Title/Abstract] OR "chordoid glioma of the third ventricle"[Title/Abstract] OR "chordoid tumor"[Title/Abstract] OR "chordoid neoplasm"[Title/Abstract] OR "suprasellar chordoid glioma"[Title/Abstract] OR "anterior third ventricle glioma"[Title/Abstract] OR "hypothalamic chordoid glioma"[Title/Abstract] OR "diencephalic chordoid glioma"[Title/Abstract] OR "chordoid glioma brain tumor"[Title/Abstract] OR "chordoid glioma CNS"[Title/Abstract] OR "chordoid glioma of the central nervous system"[Title/Abstract])*  *AND*  *("third ventricle"[Title/Abstract] OR "third ventricles"[Title/Abstract] OR "3rd ventricle"[Title/Abstract] OR "3rd ventricles"[Title/Abstract] OR "anterior third ventricle"[Title/Abstract] OR "anterior third ventricles"[Title/Abstract] OR "suprasellar region"[Title/Abstract] OR "suprasellar space"[Title/Abstract] OR hypothalamus[Title/Abstract] OR hypothalamic[Title/Abstract] OR diencephalon[Title/Abstract] OR diencephalic[Title/Abstract] OR "interventricular foramen"[Title/Abstract] OR "foramen of Monro"[Title/Abstract])* | *125* |
| *Embase* | *('chordoid glioma':ti,ab OR 'chordoid glioma of the third ventricle':ti,ab OR 'chordoid tumor':ti,ab OR 'chordoid neoplasm':ti,ab OR 'suprasellar chordoid glioma':ti,ab OR 'anterior third ventricle glioma':ti,ab OR 'hypothalamic chordoid glioma':ti,ab OR 'diencephalic chordoid glioma':ti,ab OR 'chordoid glioma brain tumor':ti,ab OR 'chordoid glioma CNS':ti,ab OR 'chordoid glioma of the central nervous system':ti,ab)*  *AND*  *('third ventricle':ti,ab OR 'third ventricles':ti,ab OR '3rd ventricle':ti,ab OR '3rd ventricles':ti,ab OR 'anterior third ventricle':ti,ab OR 'anterior third ventricles':ti,ab OR 'suprasellar region':ti,ab OR 'suprasellar space':ti,ab OR hypothalamus:ti,ab OR hypothalamic:ti,ab OR diencephalon:ti,ab OR diencephalic:ti,ab OR 'interventricular foramen':ti,ab OR 'foramen of Monro':ti,ab)* | *154* |
| *Total* | ***569*** | |

**Table S2: Quality Assessment Tool for Observational Cohort and Cross-Sectional Studies (NIH)**

| ***Study*** | **Q1** | **Q2** | **Q3** | **Q4** | **Q5** | **Q6** | **Q7** | **Q8** | **Q9** | **Q10** | **Q11** | **Q12** | **Q13** | **Q14** | **Overall rating** |
| --- | --- | --- | --- | --- | --- | --- | --- | --- | --- | --- | --- | --- | --- | --- | --- |
| Yao 2020 | Yes | Yes | Yes | Yes | No | Yes | Yes | Yes | Yes | No | Yes | NA | Yes | Yes | Fair Quality |
| Zhang 2020 | Yes | Yes | Yes | Yes | Yes | Yes | Yes | Yes | Yes | No | Yes | Yes | Yes | Yes | High Quality |
| Bielle 2015 | Yes | Yes | Yes | Yes | No | Yes | Yes | Yes | Yes | No | Yes | No | Yes | Yes | high quality |
| Huang 2024 | Yes | Yes | Yes | Yes | No | Yes | Yes | Yes | No | No | Yes | No | Yes | Yes | high quality |
| Johannes 2009 | Yes | Yes | Yes | Yes | No | Yes | Yes | Yes | Yes | No | Yes | No | Yes | Yes | high quality |

Quality Assessment Tool for Observational Cohort and Cross-Sectional Studies (NIH)

(Yes): Yes/Low risk, (No): No/High risk, (*): Unclear, NA: Not applicable

**Q1:** Was the research question or objective in this paper clearly stated?

**Q2:** Was the study population clearly specified and defined?

**Q3:** Was the participation rate of eligible persons at least 50%?

**Q4:** Were all the subjects selected or recruited from the same or similar populations (including the same time period)? Were inclusion and

exclusion criteria for being in the study prespecified and applied uniformly to all participants?

**Q5:** Was a sample size justification, power description, or variance and effect estimates provided?

**Q6:** For the analyses in this paper, were the exposure(s) of interest measured prior to the outcome(s) being measured?

**Q7:** Was the timeframe sufficient so that one could reasonably expect to see an association between exposure and outcome if it existed?

**Q8:** For exposures that can vary in amount or level, did the study examine different levels of the exposure as related to the outcome (e.g.,

categories of exposure, or exposure measured as continuous variable)?

**Q9:** Were the exposure measures (independent variables) clearly defined, valid, reliable, and implemented consistently across all study

participants?

**Q10:** Was the exposure(s) assessed more than once over time?

**Q11:** Were the outcome measures (dependent variables) clearly defined, valid, reliable, and implemented consistently across all study

participants?

**Q12:** Were the outcome assessors blinded to the exposure status of participants?

**Q13:** Was loss to follow-up after baseline 20% or less?

**Q14:** Were key potential confounding variables measured and adjusted statistically for their impact on the relationship between exposure(s)

and outcome(s)?

**Table S3: JBI Critical Appraisal Checklist for Case Reports**

| ***References*** | **Q1** | **Q2** | **Q3** | **Q4** | **Q5** | **Q6** | **Q7** | **Q8** | **Overall rating** |
| --- | --- | --- | --- | --- | --- | --- | --- | --- | --- |
| *Ki et al., 2016* | Yes | Yes | Yes | Yes | Yes | Yes | NA | Yes | High Quality |
| *Kim et al., 2010* | Yes | Yes | Yes | Yes | Yes | Yes | Yes | Yes | High Quality |
| *Kurian et al. 2005* | Yes | Yes | Yes | Yes | Yes | Yes | Yes | Yes | High Quality |
| *Lee et al., 2002* | Yes | Yes | Yes | Yes | Yes | Yes | Yes | Yes | High Quality |
| *Leeds et al., 2006* | Yes | Yes | Yes | Yes | Yes | Yes | No | Yes | High Quality |
| *Lisievici et al., 2018* | Yes | Yes | No | Yes | No | Yes | Yes | Yes | Fair Quality |
| *Liu et al., 2011* | Yes | Yes | Yes | Yes | Yes | Yes | Yes | Yes | High Quality |
| *Michotte et al., 2014* | Yes | Yes | Yes | Yes | Yes | Yes | Yes | Yes | High Quality |
| *Mohin et al., 2023* | Yes | Yes | Yes | Yes | No | No | NA | Yes | Fair Quality |
| *Morais et al., 2015* | Yes | Yes | Yes | Yes | Yes | Yes | Yes | Yes | High Quality |
| *Muthusamy et al., 2019* | Yes | Yes | Yes | Yes | Yes | No | No | Yes | Fair Quality |
| *Nakajima et al., 2003* | Yes | Yes | Yes | Yes | Yes | Yes | Yes | Yes | High Quality |
| *Nayak et al., 2025* | Yes | Yes | Yes | Yes | Yes | Yes | NA | Yes | High Quality |
| *Nga et al., 2006* | Yes | Yes | Yes | Yes | Yes | Yes | Yes | Yes | High Quality |
| *Oda et al., 2023* | Yes | Yes | Yes | Yes | Yes | Yes | Yes | Yes | High Quality |
| *Pasquier et al., 2002* | Yes | Yes | Yes | Yes | Yes | Yes | Yes | Yes | High Quality |
| *Poyuran et al., 2016* | Yes | Yes | Yes | Yes | Yes | Yes | Yes | Yes | High Quality |
| *Qixing et al., 2015* | Yes | Yes | Yes | Yes | Yes | Yes | Yes | Yes | High Quality |
| *Raizer et al., 2003* | Yes | Yes | Yes | Yes | Yes | Yes | Yes | Yes | High Quality |
| *RomeroNorojas et al., 2012* | Yes | Yes | Yes | Yes | Yes | Yes | No | Yes | High Quality |
| *Sanches et al., 2012* | Yes | Yes | Yes | Yes | Yes | Yes | Yes | Yes | High Quality |
| *CastellaNoNosanchez et al., 2000* | Yes | Yes | Yes | Yes | Yes | Yes | Yes | Yes | High Quality |
| *CastellaNoNosanchez et al., 2001* | Yes | Yes | Yes | Yes | Yes | Yes | No | Yes | High Quality |
| *Sato et al., 2003* | Yes | Yes | Yes | Yes | Yes | Yes | Yes | Yes | High Quality |
| *Scheurkogel et al., 2012* | Yes | Yes | Yes | Yes | Yes | Yes | No | Yes | High Quality |
| *Scholl et al., 2021* | Yes | Yes | Yes | Yes | Yes | Yes | No | Yes | High Quality |
| *ShiNohara et al., 2019* | Yes | Yes | Yes | Yes | Yes | Yes | Yes | Yes | High Quality |
| *Suetens et al., 2019* | Yes | Yes | Yes | Yes | Yes | Yes | Yes | Yes | High Quality |
| *Suh et al., 2003* | Yes | Yes | Yes | Yes | Yes | Yes | Yes | Yes | High Quality |
| *Takei et al., 2006* | Yes | Yes | Yes | Yes | Yes | Yes | Yes | Yes | High Quality |
| *Tanboon et al., 2014* | Yes | Yes | Yes | Yes | Yes | Yes | Yes | Yes | High Quality |
| *Taraszewski et al., 2003* | Yes | Yes | Yes | Yes | Yes | Yes | Yes | Yes | High Quality |
| *Thavaratnam et al., 2015* | Yes | Yes | Yes | Yes | Yes | Yes | Yes | Yes | High Quality |
| *Vajtai et al., 1999* | Yes | Yes | Yes | Yes | Yes | Yes | Yes | Yes | High Quality |
| *Vanhauwaert et al., 2008* | Yes | Yes | Yes | Yes | Yes | Yes | Yes | Yes | High Quality |
| *Vij et al., 2011* | Yes | Yes | Yes | Yes | Yes | Yes | No | Yes | High Quality |
| *Xian et al., 2012* | Yes | Yes | Yes | Yes | Yes | Yes | No | Yes | High Quality |
| *Yang et al., 2020* | Yes | Yes | Yes | Yes | Yes | Yes | Yes | Yes | High Quality |
| *Yao et al., 2017* | Yes | Yes | Yes | Yes | Yes | Yes | Yes | Yes | High Quality |
| *Zeinalizadeh et al., 2016* | Yes | Yes | Yes | Yes | Yes | Yes | Yes | Yes | High Quality |
| *Zhang et al., 2021* | Yes | Yes | Yes | Yes | Yes | Yes | Yes | Yes | High Quality |
| *Al zubidi 2014* | Yes | Yes | No | Yes | Yes | Yes | Yes | No | High Quality |
| *Baehring 2006* | Yes | Yes | Yes | Yes | No | No | No | No | Fair Quality |
| *Bastin 2012* | Yes | Yes | Yes | Yes | Yes | Yes | Yes | Yes | High Quality |
| *Bongetta 2015* | Yes | Yes | Yes | Yes | Yes | Yes | Yes | Yes | High Quality |
| *Bora 2015* | Yes | Yes | Yes | Yes | Yes | Yes | Yes | Yes | High Quality |
| *Brat 1998* | Yes | Yes | Yes | Yes | unclear | Yes | Yes | Yes | High Quality |
| *Buccoliero 2004* | Yes | Yes | Yes | Yes | No | Yes | Yes | No | High Quality |
| *Calanchini 2016* | Yes | Yes | Yes | Yes | Yes | Yes | Yes | Yes | High Quality |
| *Carrasco 2008* | Yes | Yes | Yes | Yes | Yes | Yes | No | Yes | High Quality |
| *Carretero 2016* | Yes | Yes | Yes | Yes | Yes | Yes | Yes | Yes | High Quality |
| *Cennachi 1999* | Yes | Yes | No | No | No | Yes | No | Yes | Fair Quality |
| *Cennachi 2001* | Yes | No | No | Yes | No | Yes | No | Yes | Fair Quality |
| *Chen 2020* | Yes | Yes | Yes | Yes | Yes | Yes | Yes | Yes | High Quality |
| *Chen 2021* | Yes | Yes | Yes | Yes | Yes | Yes | Yes | Yes | High Quality |
| *Chung 2007* | Yes | unclear | No | Yes | Yes | Yes | Yes | Yes | High Quality |
| *Cui 2020* | Yes | Yes | Yes | Yes | Yes | Yes | Yes | No | High Quality |
| *Cunha 2017* | Yes | Yes | Yes | Yes | Yes | Yes | Yes | Yes | High Quality |
| *Danilowicz 2018* | Yes | Yes | Yes | Yes | Yes | Yes | Yes | Yes | High Quality |
| *Desouza 2010* | Yes | Yes | Yes | Yes | Yes | Yes | Yes | Yes | High Quality |
| *Dias 2021* | Yes | Yes | Yes | Yes | Yes | No | No | No | High Quality |
| *Dogan 2018* | Yes | Yes | Yes | Yes | Yes | Yes | Yes | Yes | High Quality |
| *Dziurzynski 2009* | Yes | Yes | Yes | Yes | Yes | Yes | Yes | Yes | High Quality |
| *Erwood 2017* | Yes | Yes | Yes | Yes | Yes | Yes | Yes | No | High Quality |
| *Estronza 2018* | Yes | Yes | Yes | Yes | Yes | Yes | Yes | Yes | High Quality |
| *Feng 2025* | Yes | Yes | Yes | Yes | Yes | Yes | Yes | Yes | High Quality |
| *Galloway 2001* | Yes | Yes | Yes | Yes | Yes | Yes | No | Yes | High Quality |
| *GarcíaNogarcía 2017* | Yes | Yes | Yes | Yes | Yes | Yes | Yes | Yes | High Quality |
| *Ghosal 2012* | Yes | Yes | Yes | Yes | Yes | Yes | Yes | Yes | High Quality |
| *Grand 2002* | Yes | Yes | Yes | Yes | No | Yes | No | Yes | High Quality |
| *Hanbali 2001* | Yes | Yes | Yes | Yes | Yes | Yes | Yes | Yes | High Quality |
| *Hewer 2015* | Yes | Yes | Yes | Yes | No | No | No | No | Fair Quality |
| *Hinai 2011* | Yes | Yes | Yes | Yes | Yes | Yes | No | Yes | High Quality |
| *Hisao 2000* | Yes | Yes | Yes | Yes | Yes | Yes | No | Yes | High Quality |
| *Horbinski 2009* | Yes | unclear | Yes | Yes | unclear | Yes | Yes | Yes | High Quality |
| *Hung 2021* | Yes | Yes | Yes | Yes | Yes | No | No | Yes | High Quality |
| *Huo 2018* | Yes | Yes | Yes | Yes | Yes | Yes | No | Yes | High Quality |
| *Iwami 2009* | Yes | Yes | Yes | Yes | Yes | Yes | No | Yes | High Quality |
| *Jain 2008* | Yes | Yes | Yes | Yes | No | No | No | No | Fair Quality |
| *Jung 2006* | Yes | Yes | Yes | Yes | Yes | Yes | Yes | Yes | High Quality |
| *Kawasaki 2009* | Yes | Yes | Yes | No | No | No | Yes | Yes | High Quality |
| *Can 2012* | Yes | Yes | Yes | Yes | Yes | Yes | No | Yes | High Quality |
| *Destefani 2015* | Yes | Yes | Yes | No | No | No | Yes | Yes | High Quality |

(Yes): Yes, (No): No, (*): Unclear, Not/Applicable (NA)

**Q1. Were patient’s demographic characteristics clearly described?**

Does the case report clearly describe patient's age, sex, race, medical history, diagnosis, prognosis, previous treatments, past and current diagnostic test results, and medications? The setting and context may also be described.

**Q2. Was the patient’s history clearly described and presented as a timeline?**

A good case report will clearly describe the history of the patient, their medical, family and psychosocial history including relevant genetic information, as well as relevant past interventions and their outcomes.

**Q3. Was the current clinical condition of the patient on presentation clearly described?**

The current clinical condition of the patient should be described in detail including the uniqueness of the condition/disease, symptoms, frequency and severity. The case report should also be able to present whether differential diagnoses was considered.

**Q4. Were diagnostic tests or methods and the results clearly described?**

A reader of the case report should be provided sufficient information to understand how the patient was assessed. It is important that all appropriate tests are ordered to confirm a diagnosis and therefore the case report should provide a clear description of various diagnostic tests used (whether a gold standard or alternative diagnostic tests). Photographs or illustrations of diagnostic procedures, radiographs, or treatment procedures are usually presented when appropriate to convey a clear message to readers

**Q5. Was the intervention(s) or treatment procedure(s) clearly described?**

It is important to clearly describe treatment or intervention procedures as other clinicians will be reading the paper and therefore may enable clear understanding of the treatment protocol. The report should describe the treatment/intervention protocol in detail; for e.g. in pharmacological management of dental anxiety No the type of drug, route of administration, drug dosage and frequency, and any side effects.

**Q6. Was the post intervention clinical condition clearly described?**

A good case report should clearly describe the clinical condition post intervention in terms of the presence or lack thereof symptoms. The outcomes of management/treatment when presented as images or figures would help in conveying the information to the reader/clinician.

**Q7. Were adverse events (harms) or unanticipated events identified and described?**

With any treatment/intervention/drug, there are bound to be some adverse events and, in some cases, they may be severe. It is important that adverse events are clearly documented and described, particularly when a new or unique condition is being treated or when a new drug or treatment is used. In addition, unanticipated events, if any that may yield new or useful information should be identified and clearly described.

**Q8. Does the case report provide takeaway lessons?**

Case reports should summarize key lessons learned from a case in terms of the background of the condition/disease and clinical practice guidance for clinicians when presented with similar cases.

**Table S4: JBI Critical Appraisal Checklist for Case Series**

| ***References*** | **Q1** | **Q2** | **Q3** | **Q4** | **Q5** | **Q6** | **Q7** | **Q8** | **Q9** | **Q10** | **Overall rating** |
| --- | --- | --- | --- | --- | --- | --- | --- | --- | --- | --- | --- |
| *Kobayashi et al., 2013* | Yes | Yes | Yes | Yes | Yes | Yes | Yes | Yes | Yes | No | High Quality |
| *KoNovalov et al., 2023* | Yes | Yes | Yes | Yes | Yes | Yes | Yes | Yes | Yes | Yes | High Quality |
| *Ni et al., 2013* | Yes | Yes | Yes | NA | Yes | Yes | Yes | No | Yes | No | Fair Quality |
| *Oh et al. 2016* | Yes | Yes | Yes | NA | Yes | No | Yes | No | Yes | No | Low Quality |
| *Reifenberger et al., 1999* | Yes | Yes | Yes | NA | Yes | Yes | Yes | Yes | Yes | No | High Quality |

(Yes): Yes, (No): No, (*): Unclear, Not/Applicable (NA)

**Q1. Were there clear criteria for inclusion in the case series?**

The authors should provide clear inclusion (and exclusion criteria where appropriate) for the study participants. The inclusion/exclusion criteria should be specified (e.g., risk, stage of disease progression) with sufficient detail and all the necessary information critical to the study.

**Q2. Was the condition measured in a standard, reliable way for all participants included in the case series?**

The study should clearly describe the method of measurement of the condition. This should be done in a standard (i.e. same way for all patients) and reliable (i.e. repeatable and reproducible results) way.

**Q3. Were valid methods used for identification of the condition for all participants included in the case series?**

Many health problems are not easily diagnosed or defined and some measures may not be capable of including or excluding appropriate levels or stages of the health problem. If the outcomes were assessed based on existing definitions or diagnostic criteria, then the answer to this question is likely to be yes. If the outcomes were assessed using observer reported, or self-reported scales, the risk of over or under reporting is increased, and objectivity is compromised. Importantly, determine if the measurement tools used were validated instruments as this has a significant impact on outcome assessment validity.

**Q4. Did the case series have consecutive inclusion of participants?**

Studies that indicate a consecutive inclusion are more reliable than those that do not. For example, a case series that states ‘we included all patients (24) with osteosarcoma who presented to our clinic between March 2005 and June 2006’ is more reliable than a study that simply states, ‘we report a case series of 24 people with osteosarcoma.’

**Q5. Did the case series have complete inclusion of participants?**

The completeness of a case series contributes to its reliability (1). Studies that indicate a complete inclusion are more reliable than those that do not. A stated above, a case series that states ‘we included all patients (24) with osteosarcoma who presented to our clinic between March 2005 and June 2006’ is more reliable than a study that simply states ‘we report a case series of 24 people with osteosarcoma.’

**Q6. Was there clear reporting of the demographics of the participants in the study?**

The case series should clearly describe relevant participant’s demographics such as the following information where relevant: participant’s age, sex, education, geographic region, ethnicity, time period, education.

**Q7. Was there clear reporting of clinical information of the participants?**

There should be clear reporting of clinical information of the participants such as the following information where relevant: disease status, comorbidities, stage of disease, previous interventions/treatment, results of diagnostic tests, etc.

**Q8. Were the outcomes or follow-up results of cases clearly reported?**

The results of any intervention or treatment should be clearly reported in the case series. A good case study should clearly describe the clinical condition post no intervention in terms of the presence or lack of symptoms. The outcomes of management/treatment when presented as images or figures can help in conveying the information to the reader/clinician. It is important that adverse events are clearly documented and described, particularly a new or unique condition is being treated or when a new drug or treatment is used. In addition, unanticipated events, if any that may yield new or useful information should be identified and clearly described.

**Q9. Was there clear reporting of the presenting site(s)/clinic(s) demographic information?**

Certain diseases or conditions vary in prevalence across different geographic regions and populations (e.g. women vs. men, sociodemographic variables between countries). The study sample should be described in sufficient detail so that other researchers can determine if it is comparable to the population of interest to them.

**Q10. Was statistical analysis appropriate?**

As with any consideration of statistical analysis, consideration should be given to whether there was a more appropriate alternate statistical method that could have been used. The methods section of studies should be detailed enough for reviewers to identify which analytical techniques were used and whether these were suitable.
